# Estimation of Knee Joint Angle from Surface EMG Using Multiple Kernels Relevance Vector Regression

**DOI:** 10.3390/s23104934

**Published:** 2023-05-20

**Authors:** Hui-Bin Li, Xiao-Rong Guan, Zhong Li, Kai-Fan Zou, Long He

**Affiliations:** 1 School of Mechanical Engineering, Nanjing University of Science and Technology, Nanjing 210094, China; lhb1011040110@njust.edu.cn (H.-B.L.); zhong0814@njust.edu.cn (Z.L.); zoukaifan@njust.edu.cn (K.-F.Z.); helong_zyy@163.com (L.H.); 2 Zhiyuan Research Institute, Hangzhou 310013, China

**Keywords:** motion intention, surface electromyography (sEMG), joint angle estimation

## Abstract

In wearable robots, the application of surface electromyography (sEMG) signals in motion intention recognition is a hot research issue. To improve the viability of human–robot interactive perception and to reduce the complexity of the knee joint angle estimation model, this paper proposed an estimation model for knee joint angle based on the novel method of multiple kernel relevance vector regression (MKRVR) through offline learning. The root mean square error, mean absolute error, and R2_score are used as performance indicators. By comparing the estimation model of MKRVR and least squares support vector regression (LSSVR), the MKRVR performs better on the estimation of the knee joint angle. The results showed that the MKRVR can estimate the knee joint angle with a continuous global MAE of 3.27° ± 1.2°, RMSE of 4.81° ± 1.37°, and R^2^ of 0.8946 ± 0.07. Therefore, we concluded that the MKRVR for the estimation of the knee joint angle from sEMG is viable and could be used for motion analysis and the application of recognition of the wearer’s motion intentions in human–robot collaboration control.

## 1. Introduction

Wearable robots as electromechanical integration systems, closely cooperating with the human body, need to recognize the human intention and finally complete actions in collaboration with human joints [1]. The human–robot interaction (HRI) system performs an important role in the human–robot collaborative control of wearable robots, and the occurrence of new human–robot interactive methods requires higher perceptive capabilities of wearable robots than before, but it is difficult for wearable robots to understand human intentions in advance and assist them in completing collaborative tasks. The recognition methods based on biological signals are expected to realize more effective human motion intention understanding in recent years [2]. Biological signals are often generated by human organs before the execution of human actions, which shows a certain degree of motion predictability [3]. Consequently, the human–robot interaction, based on surface electromyography (sEMG) signals, has attracted a great deal of special attention. The HRI system with sEMG signals can realize a natural control similar to brain control and can be accepted by the wearer easily [4]. The innovative wearable real-time Human Activity Recognition (HAR) system that integrates biosensors into the knee bandage has been proposed, which shows that the knee bandage is a potential wearable sensor carrier of sEMG sensors (and other types of wearables) to measure knee joints [5,6]. The sEMG signals are generated by the contraction of the skeletal muscle, which is a sequence of potential actions issued by multiple motor units and the results of comprehensive superposition on the skin surface in terms of time and space. The sEMG signals generate 30 ms to 150 ms ahead of the schedule of human limb movements, so the sEMG signals can be extracted through electrodes attached to the skin surface and regarded as the activated signals to estimate the limb movements in advance, and they can make human motion intention recognition more timely and more accurate. The knee joint angle is the most significant parameter that affects lower limb movements. While walking and running, the amplitude and speed of changes in knee joint angle are significant, which can represent the bending or stretching state of the lower limbs and, thus, reflect the human motion intention. Compared to the hip and the ankle, knee motion can be considered as a single degree of freedom flexion/extension motion, and its angle prediction is relatively easy to achieve. The sEMG signals are highly nonlinear and susceptible to interference, so it is difficult to establish the mapping relationship between the sEMG signals and the knee joint angle for estimating the intention of the lower limb movements. Furthermore, the performance of motion intention estimation directly affects the wearable robot’s ability to collaborate with humans.

As presented in many recent studies, the joint angle is the main indicator of movement estimation, depending on sEMG, and it has always been applied in wearable robots. However, the technology has been developed, and mapping is difficult to construct between the joint angle and sEMG signals. The control system of most wearable robots requires continuous control signals to realize real-time control and to follow and match the wearer’s movements. In general, how to extract accurate motion intention from sEMG and how to predict joint movements in advance are still thorny problems for wearable robots up to now. However, since the lower limb muscles are presented deep beneath the skin with significant overlap among them, the estimation of the sEMG signals from such muscles is more challenging when compared to the upper limb muscles [7]. The estimation of the knee joint angle from sEMG signals captured from lower limb muscles is more challenging because the flexion and extension of the knee joint are caused by multiple muscles in nature, and it is rather difficult to eliminate the crosstalk issue caused by the physiological differences in the active muscles.

Many studies have explored the relationship between sEMG and joint movement, as well as the methods of accurate estimation of the joint motion from sEMG signals. In [8], the researchers composed the AdaBoost and random forests (RFs) as new approaches for estimating motion intentions based on the sEMG, and they studied the performance of the execution time and the estimation accuracy. In [9,10], to improve the estimation accuracy of the knee joint angle and to reduce the estimation artifacts, researchers proposed fusion-based algorithms based on the Kalman filter. In [11], the researchers compared the motion recognition performance of lower limbs with Gaussian kernel-based linear discriminant analysis (LDA) and support vector machines (SVMs) by fused sEMG signals and accelerometer signals. In [12,13], researchers used least-squares support vector regression (LSSVR) to predict human lower limb periodic motions from multi-channel sEMG signals. In [14], researchers presented deep-recurrent neural networks (DRNN) by fusing sEMG and kinematics signals to predict the knee joint angle in real-time. In [15], the researchers proposed the deep learning approach to predict the foot–floor contact signals in more natural walking conditions based on sEMG. In [16], researchers established a mapping relationship between the sEMG signals and the knee joint angle by using the wavelet neural network and estimating the knee joint angle based on the sEMG signals from the vastus rectus (VR). In [17], the researchers proposed a multiple linear regression model to predict the knee joint moment by constructing the mapping model of the EMG signals and knee joint angle during the extension–flexion movements. In [18], researchers fused the transfer-learning and long-term recurrent convolution network to predict knee joint angle. In [19], researchers presented the improved principal component analysis algorithm, based on the kernel method, to reduce the dimension of the sEMG dataset in the process of predicting the knee joint angle.

In addition to the above research on the mapping relationship between knee joint angle and sEMG signals for knee movement estimation, researchers also carried out studies on the mechanism of muscle action and knee joint movement. In [20], the researchers acquired sEMG signals of semitendinosus, biceps femoris, rectus femoris, vastus medialis, vastus lateralis, gastrocnemius, tibialis anterior, and knee joint angle measurement with a goniometer from 20 able-bodied subjects so as to estimate knee range of motion. In [21], researchers captured sEMG signals of the vastus medialis, rectus femoris, biceps femoris, and the three-dimensional kinematics of lower extremity joints from four participants to estimate the continuous knee joint angle. In [22], researchers constructed the mapping relationship between the sEMG signals of biceps femoris, vastus medialis, rectus femoris, semitendinosus, and the continuous knee joint angle. In [23], researchers constructed the musculoskeletal biomechanical model to connect the sEMG signals and the knee joint torque. In [4], researchers built the mapping model between the activation coefficients and the knee joint angle based on muscle synergy theory and a generalized regression neural network. In [24], researchers estimated the knee joint angle in voluntary muscle contraction and the functional electrical stimulation-induced contraction of knee joint extension motion. In [25], researchers approximated the active joint moments of the subjects during the swing phase. In [26], researchers proposed two single-joint active training strategies to estimate the single-joint voluntary motion intention, based on the sEMG signals.

However, the lack of detailed sEMG characteristics for motion recognition has been a difficult issue with regards to developing safe and intuitive interactions with robots [27]. Furthermore, to reduce the influence of highly nonlinear and susceptible sEMG signals on estimation performance, the average feature (AF) is evolved to process the sEMG signals. The average feature values of sEMG signals were used as the input, and the knee joint angle was used as the output in this study. Multiple kernel relevance vector regression (MKRVR) was used to estimate the knee joint angles. Last, but not least, MATLAB was used to evaluate the accuracy and to verify the feasibility of the estimation model.

This paper aims to recognize human lower limb motion intention using sEMG signals and knee joint angle. To reduce the effects of different gait speeds and strides among various subjects in the data collection process, the gait cycle was used to measure the sample data. All of the walking data of each subject were divided into several gait cycles (GCs) for consistency in the sample size between the sEMG signals and the knee joint angle. The AF of sEMG signals in each GC was calculated to ensure sample size consistency and to decrease interference between different sample channels. After signal processing, a mapping model between the sEMG signals and the knee joint angle was constructed to represent the relationship between the muscle actions and the knee movements, based on MKRVR. The comparison between MKRVR and other regression models, such as standard RVR and LSSVR, indicated that the MKRVR has strong applicability in estimating the knee joint angle. In addition, it can improve angle estimation accuracy and smoothness. Notably, the MKRVR performed better in small-sample regression prediction. The proposed method has the potential to enhance offline intention prediction and to facilitate rapid prediction of human intentions in HRI systems, based on sEMG signals.

## 2. Materials

### 2.1. Data Collection

**Figure 1:**
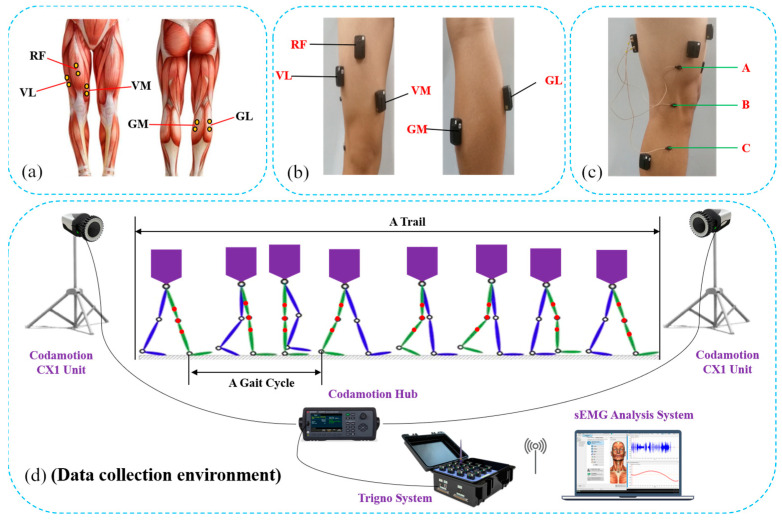
The schematic of the experiment; (**a**,**b**) Position of the sEMG sensor patch electrodes; (**c**) Layout of Markers A, B, and C; (**d**) Data collection environment.

To obtain the sEMG signals samples of human knee joint movements, this experiment acquired sEMG signals by using the Trigno Wireless sEMG instrument, and a three-dimensional motion capture system, called Codamotion, was used to acquire kinematic data regarding the knee joint. The sampling frequency of sEMG signals is 2000 Hz, and the sampling frequency of knee joint angles is 100 Hz. To ensure the sEMG signals and the knee joint angle with the same sample frequency, Codamotion is connected to the Trigno system. The experimental device is arranged, as shown in Figure 1.

In the experiment, five healthy male participants, without any history of neuromuscular disorders and muscular atrophy or lower extremity surgery, participated in the experiment (age 24.2 ± 1.6 years, height 181 ± 3.8 cm, mass 72.5 ± 6.9 kg). Their information was listed in Table 1. The subjects were randomly selected among the volunteer students of Nanjing University of Science and Technology and were represented as S1 to S5. The experimental scheme was approved by the Human Ethics Review Committee of Nanjing University of Science and Technology.

Before the collection of the sEMG signals and the knee joint angle, the experimental protocol was introduced to all subjects, and informed consent was given. All subjects were required to keep calm to avoid affecting the measurement results. When collecting the sEMG signals, the first step was to determine the muscle positions by palpation. The second step was to scrape off the hair on the sticking position and to polish the dead skin by using sandpaper. The third step was to wipe and to disinfect the skin with alcohol to ensure the stability of the sEMG signals and to reduce noise interference. Finally, the sEMG electrode was stuck on the surface of the test muscle. Before the start of the experiment, the subject was asked to calm down for 30 min, continuously, after preparing the experiment device. After collecting the sEMG signals, the subject walked back and forth nearly 20 times in the span of 15 min along a straight line about 5 m in length. The walking frequency of the subject must be his daily gait. All the sEMG signals from different subjects were collected in the morning. There were approximately 60 full GCs, and corresponding sEMG signals were recorded from each subject in total.

From research based on the clinical information and experiential practice, there are nine muscles, up and down the knee joint, affecting knee movements. In [4], researchers selected the vastus rectus muscle (VR), vastus lateralis muscle (VL), semitendinosus muscle (SM), biceps muscle (BM), tibialis anterior muscle (TA), extensor pollicis longus (EP), and gastrocnemius muscle (GM) to acquire the sEMG signals of lower limb movements. According to [28,29], to reduce the crosstalk between different sample channels, four to eight muscles are usually used to extract the sEMG signals. Consequently, in this experiment, the rectus femoris (RF), vastus medialis (VM), vastus lateralis (VL), gastrocnemius medialis (GM), and gastrocnemius lateralis (GL) were selected to acquire the sEMG signals around the knee joint and to represent the flexion and extension movements of the knee joint. RF is located on the front of the thigh, above the middle of the knee joint. It is the major muscle that generates the flexion and extension movements of the knee joint. VL and VM are located on both sides of the thigh, and GL and GM are located on both sides of the leg. These four muscles affect the stability of the knee joint in the state of flexion and extension. The position of the sEMG sensor patch electrodes is shown in Figure 1a,b. To obtain the knee joint angle, three markers were evenly distributed on the upper and lower sides of the knee joint. They were located on the same line in the sagittal plane of the human body. As shown in Figure 1c, Marker B is placed at the approximate center of the knee joint. Marker A and Marker C were placed on the projection line of the femur and tibia on the sagittal plane, respectively. The knee joint angle can be calculated according to the spatial motion trajectory of Markers A, B, and C.

### 2.2. Data Processing

The raw sEMG signals include noise and a large amount of data with DC offset and motion artifacts or aliasing [30]. Before applying for angle estimation, these raw sEMG signals must be filtered from 20 Hz to 500 Hz by using a fourth-order recursive Butterworth bandpass filter. The cutoff frequency is 20 Hz. After the bandpass is filtered, the full-wave rectification was continued. Finally, the sEMG signals were filtered from 5 Hz to 10 Hz by using a fourth-order recursive Butterworth low-pass filter to smooth the signals. The cutoff frequency was 6 Hz. The sampling frequency of the sEMG signals was much higher when compared to the knee joint angle. Therefore, the preprocessed sEMG data were resampled to 100 data points, according to the AF method. The sEMG and knee joint angle data were not involved in the continuous signals, corresponding to the transition phase.

In this study, the sEMG signals were not imported directly into the estimation model. Instead, the amplitude was extracted from the sEMG signals by using a low-pass filter directly. Due to the feature extraction in the time domain, requiring no transformation and fewer calculations, the amplitude was selected as the input feature to estimate the knee joint angle, and it was represented by *x*. It was proposed to calculate the average value of the sEMG feature signal to reduce the influence caused by the sample difference and the interference between different sample channels. After filtering, amplification, and extraction, the sEMG signals were normalized with maximum and minimum amplitudes, as Equation (1) shows.

(1) $$x_{i}\left(j\right)=\frac{x_{i}\left(j\right)-\min\left(\left\{x_{i}\left(j\right)\right\}\right)}{\max\left(\left\{x_{i}\left(j\right)\right\}\right)-\min\left(\left\{x_{i}\left(j\right)\right\}\right)}$$

The AF employed in this paper is presented as follows:

(2) $$\mathrm{AF}=\frac{\sum_{j=1}^{M}x_{i}\left(j\right)}{\left(M-1\right)\Delta t}$$

where *x_i_*(*j*) describes the *j*th sEMG signal in the *i*th sliding windows. AF represents the average value of the sEMG feature signal. *M* represents the number of sliding window feature signals within a period and is equal to 10. Δ*t* is the sampling period and is equal to 1/2000.

The raw kinematic data of the knee joint collected from Markers A, B, and C were coordinate points, but 2 vectors can be obtained from Markers A, B, and C, and these 2 vectors are $V_{AB}$ and $V_{BC}$. Furthermore, the knee joint angle can be calculated, as follows:

(3) $$\varphi_{knee}=\arccos\left(\frac{V_{AB}\cdot V_{BC}}{\left|V_{AB}\right|\left|V_{BC}\right|}\right)$$

In this study, to reduce the error caused by multiple sets of data concatenation in motion estimation, the gait cycle was introduced as the unit to measure the sample sizes of different subjects, and the maximum sample sizes of each subject ranged from 63 GCs to 82 GCs. One GC of different subjects with a different number of sample points was considered. GCs are listed in Table 2, and S is represented as the subject, and n is represented as the sample size.

After processing the raw data of the sEMG signals and knee joint angle, the datasets of the estimation model can be established, which are $\left[{\mathrm{AF}}_{t},Y_{t}\right]$, where the sEMG ${\mathrm{AF}}_{t}$ is the input, and the knee joint angle $Y_{t}=\varphi_{knee,t}$ is the output. In this work, the maximum dataset size was selected as 60 GCs.

## 3. Methods

Due to the complexity of the sEMG signals extracted from different subjects, it is difficult to establish a general mathematical model to describe the mapping relationship between the sEMG signals and the knee joint angle. In addition, the biomechanical model of the relationship between the sEMG and the knee joint angle has a long way to go in practical application. Therefore, a novel model-free estimation method has been proposed, depending on the machine learning algorithm to establish a universal estimation model between the sEMG signals and knee joint angle with the evolution function. In this paper, the relevance vector machine (RVM) is used to establish the mapping model between the sEMG signals and the knee joint angle. Before the beginning of the training process, the sEMG dataset was used as the input dataset, which is divided into the training data and the testing data. The output dataset is the knee joint angle. After the training process, the regression model was established. It estimates the knee joint angle, according to the muscle action in advance, based on the mapping relationship between the sEMG signals and the knee joint angle.

### 3.1. Estimation Model Based on Multiple Kernel Relevance Vector Regression

The RVM is a machine learning algorithm that integrates kernel function, Bayesian learning, and automatic correlation decision mechanisms. It can achieve high-precision characterization of the sparse probability distribution of the high-dimensional space separation of sample data. This method is suitable for small sample data regression and classification tasks. It provides the advantages of flexible kernel function selection and simple model parameter setting. The basic idea is that the small sample data are mapped to a high-dimensional feature space, based on kernel functions. A small number of relevant vectors that accurately characterize the sparse distribution of sample data are obtained using Bayesian learning and an automatic correlation decision mechanism to improve the data generalization performance [31,32]. The relevance vector regression (RVR) model is a linear regression model using the kernel function as the basis function. The relevant parameters have independently prior distributions [33]. They are mathematically described as follows.

Let ${\left\{x\right\}}_{u=1}^{N}$ and ${\left\{t\right\}}_{u=1}^{N}$ be the input and output vectors, respectively, and the objective *t* can be obtained using the regression model, shown in Equation (4).

(4) $$t=y\left(x\right)+\xi_{n}$$

where $\xi_{n}$ is the noise with zero mean and variance $\sigma^{2}$, with *y*(*x*) defined as:

(5) $$y\left(x\right)=\sum_{u=1}^{N}w_{u}K\left(x,x_{u}\right)+w_{0}$$

where $K\left(x,x_{u}\right)$ is the kernel function, $w_{u}$ is the weight, and $w_{0}$ is the deviation.

To address the problem of poor generalization performance of regression on small samples of sEMG signals while walking, a knee joint angle regression model was proposed, which is based on multiple kernel RVR (MKRVR). The model effectively uses multiple kernel functions (RVR), which can explore more nonlinear features, containing knee motion difference information in high-dimensional feature space, thus improving the learning performance of RVR to accurately characterize the sparse distribution of knee motion difference, as well as improving the regression generalization performance for small sample data. Compared with the single kernel RVR, MKRVR can process nonlinear data by mapping data to high-dimensional space, while the single kernel RVR can only process linear separable data [34]. In addition, MKRVR can also use multiple kernel functions to process different types of data to improve the regression performance and accelerate the training process through parallel computing to increase the training efficiency [35]. MKRVR can largely combine the advantages of polynomial, Gaussian, and sigmoid kernel functions. This achieves a better capability of generalization and obtains predicted results [36]. The MKRVR uses three kernel functions to construct the regression model: the Gaussian RBF kernel, the polynomial kernel, and the sigmoid kernel, respectively. The kernel function is calculated, as shown in the following equation:

(6) $$\{\begin{array}{c} K_{gaussian}\left(x,z\right)=\exp\left(-\gamma {\left\|x-z\right\|}^{2}\right)\\ K_{polynomial}\left(x,z\right)={\left(\gamma x^{T}z+c\right)}^{d}\\ K_{sigmoid}\left(x,z\right)=\tanh\left(\gamma x^{T}z+c\right)\\ K_{MK}=0.8K_{gaussian}+0.17K_{polynomial}+0.03K_{sigmoid} \end{array}$$

Here, γ, *c*, and *d* are the kernel parameters, *K_gaussian_* is the Gaussian RBF kernel, *K_polynomial_* is the polynomial kernel, and *K_sigmoid_* is the sigmoid kernel.

**Figure 2:**
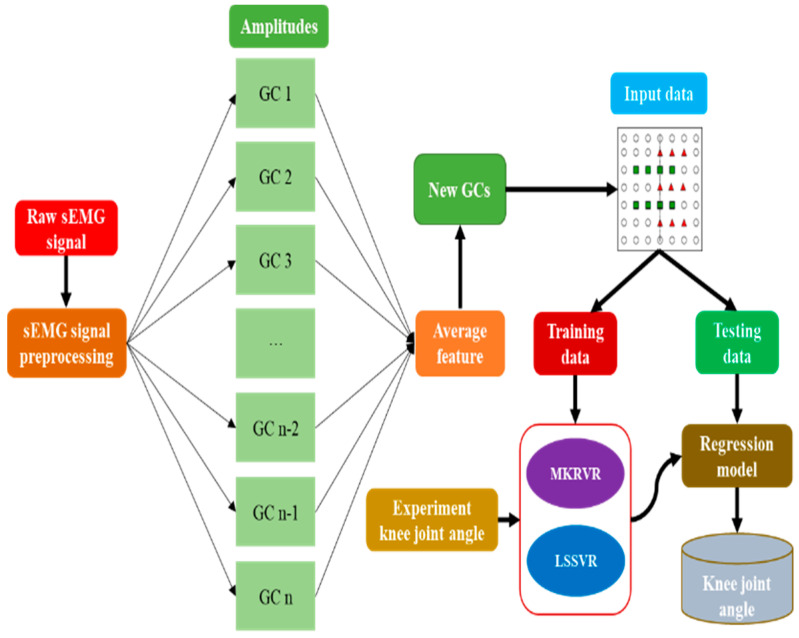
The estimation process of the proposed method.

In this study, the estimation model was proposed, according to the MKRVR. The process of estimation is shown in Figure 2. It can be seen, in the figure, that the amplitudes of sEMG signals were extracted, based on the gait cycle. The average features were calculated to stack the sEMG data of different gait cycles. The comparison with the LSSVR was used to illustrate the advantages and the advancement of the MKRVR for joint angle estimation, depending on small sample sEMG data. In the process, the sEMG signals of human walking were divided into several GCs. The number of sample points in each GC is different. The sEMG frequency of each GC is 2000 Hz, and the knee joint angle frequency of each GC is 100 Hz. Thus, the AF was used to resample the number of sEMG signals in each GC as equal to the number of knee joint angles and to stack the sEMG data of different GCs. Finally, the sEMG signal was composed, and the corresponding knee joint angle was used as the input and output to construct the mapping model between sEMG signals and the knee joint angle, based on MKRVR and LSSVR.

### 3.2. Parameter Sets

In this study, the key parameters are the kernel parameters and kernel weight of multiple kernels. The kernel parameters are the degree of the polynomial kernel (*d*), the width of the kernel (γ), and the offset of the kernel (*c*), respectively. The kernel weights of multiple kernels are the weight of the Gaussian RBF kernel (*K_gaussian_*), the weight of the polynomial kernel (*K_polynomial_*), and the weight of the sigmoid kernel (*K_sigmoid_*), respectively. The parameters for the algorithm were set, as recorded in Table 3.

### 3.3. Performance Indicators

In this study, the mean absolute error (MAE), root mean square error (RMSE), and R2_score (R^2^) are the performance indicators. MAE is the result of averaging the absolute value of the difference between each true value and each predicted value. MAE reflects the overall difference between the predicted and true values of the samples. Furthermore, MAE reflects the overall situation of all samples of a model, and if there are obvious outliers, it will disturb the results more. To consider the influence of outliers, the RMSE is introduced, and the absolute value is replaced by the square, which amplifies the influence of outliers. To exclude the influence of the scale of the dataset and to portray the performance of the regression model, R^2^ was adopted to evaluate the accuracy of the model. These factors are defined as follows. Unless specified otherwise, in these equations below, *n* is the sample size, $y_{i}$ is the true value of the *i*th sample, and ${\hat{y}}_{i}$ is the predicted value of the *i*th sample.

(1) Mean absolute error (MAE):

(7) $$\mathrm{MAE}=\frac{1}{n}\sum_{i=1}^{n}\left|{\hat{y}}_{i}-y_{i}\right|$$

(2) Root mean square error (RMSE):

(8) $$\mathrm{RMSE}=\sqrt{\frac{1}{n}\sum_{i=1}^{m}\left(y_{i}-{\hat{y}}_{i}\right)}$$

The range of MAE and RMSE is [0, +∞), and, when the predicted value is completely consistent with the true value, it is equal to 0. The larger the error, the larger the value. The smaller the value, the better the accuracy of the estimation model.

(3) R2_score (R^2^):

(9) $$R^{2}=1-\frac{\sum_{i=1}^{n}{\left(y_{\mathrm{true}}^{\left(i\right)}-y^{\left(i\right)}\right)}^{2}}{\sum_{i=1}^{n}{\left(y_{\mathrm{mean}}^{\left(i\right)}-y^{\left(i\right)}\right)}^{2}}$$

where $y_{\mathrm{true}}^{\left(i\right)}$ is the true value, $y^{\left(i\right)}$ is the predicted value, and $y_{\mathrm{mean}}^{\left(i\right)}$ is the average of true values. The closer the R2_score is to 1, the better the estimation. R2_score = 0 means the predicted value is equal to the average value, and the training model is not ideal. R2_score < 0 means the model is not as effective as simply taking the average value.

## 4. Results

This study requires finding a novel and rapid method for estimating the knee joint angle. Validate the model and analyze the impact of training data on the model by selecting multiple gait cycles to form a training dataset of different sample sizes. Take the sample of 1 GC as the initial training data. Each time the sample size changes, five GCs are added. A total of 13 training datasets of different sizes are set. The sample size range is 1 to 60 GCs. First of all, to determine the performance of MKRVR, a comparison between MKRVR and other methods is proposed. The contrast methods are the standard RVR with Gaussian RBF kernel, LSSVR, random forest (RF), and random forest with principal component analysis (RFPCA). In the RF model [37], the number of trees in the forest (*N*), the number of features of the input (*Nf*), and the minimum size of the terminal nodes (*Nm*) were determined, and they are set as *N* = 50, *Nf* = 5, and *Nm* = 5.

The results are listed in Table 4. The results showed that the R2_Score of MKRVR is slightly better than RVR. The average RMSE and MAE of MKRVR is better than RVR, but the average running time of MKRVR is less than RVR. Considering the quality of the dataset and the setting of the hyperparameter, it can be considered that the performance of MKRVR is superior to standard RVR with a Gaussian RBF kernel. In contrast to RF and RFPCA, although the R2_Score, RMSE, and MAE of RFPCA are better than RF, RVR, and LSSVR, the R2_Score, RMSE, and MAE of RVR are better than RF. The R2_Score, RMSE, and MAE of MKRVR are better than RFPCA. MKRVR has more advantages in estimating running time than RF/RFPCA/LSSVR. Different regression methods have different advantages in different problems because the SVR and RVR are similar, in principle. RVR is developed on the basis of SVR. Thus, the subsequent analysis focused on the comparison between improved RVR (MKRVR) and improved SVR (LSSVR).

**Figure 3:**
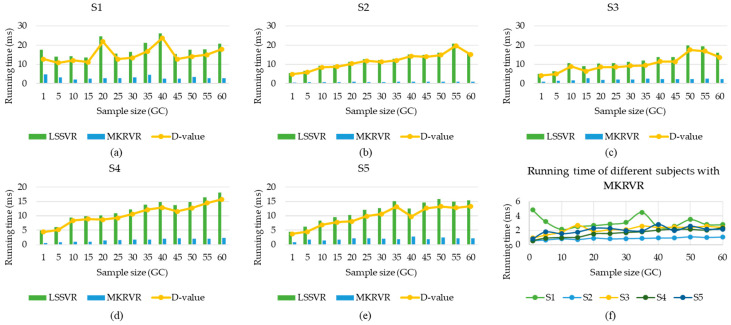
The relative running time comparison for the estimation with LSSVR and MKRVR.

**Figure 4:**
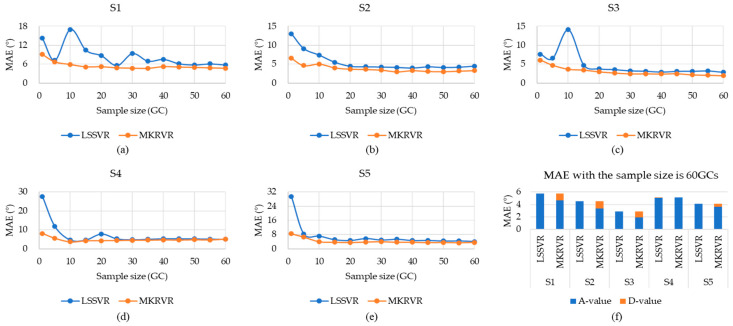
The MAE comparison for the estimation with LSSVR and MKRVR.

**Figure 5:**
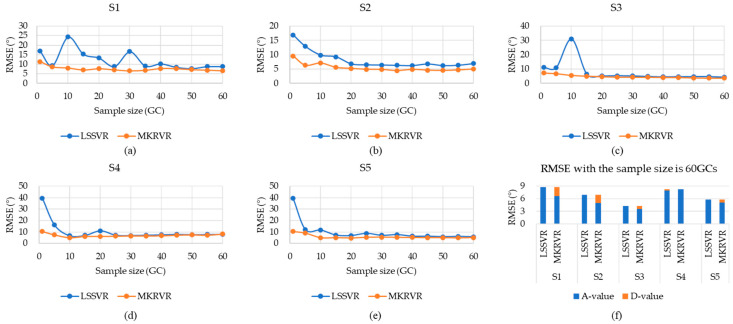
The RMSE comparison for the estimation with LSSVR and MKRVR.

**Figure 6:**
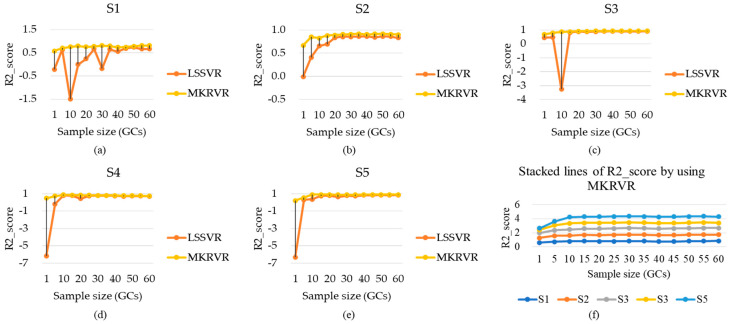
The R2_score comparison for the estimation with LSSVR and MKRVR.

**Figure 7:**
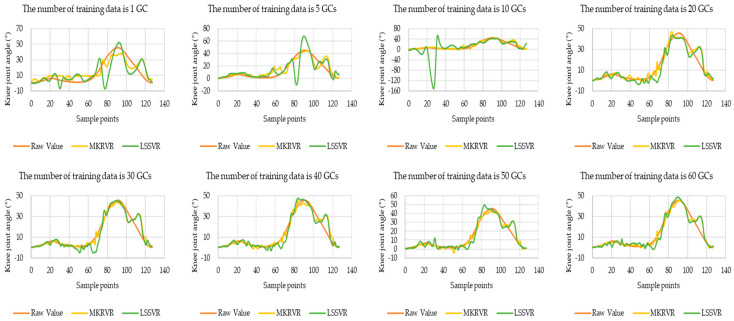
Results for estimation with different sample sizes of Subject 3.

Therefore, the results of LSSVR and MKRVR are compared to illustrate the proposed method. The test running time and estimation accuracy were presented to verify the advantages and performance of the estimation model. The results have been illustrated in Figure 3, Figure 4, Figure 5, Figure 6 and Figure 7. The comparison results of running time, MAE, and RMSE for different methods are listed in Table 5.

Figure 3 shows the different running times of different subjects using LSSVR and MKRVR to estimate the knee joint angle. Figure 3a–e used a histogram to compare the running time of different methods for estimating the knee joint angle and the yellow broken line, which represents the difference (D-value) of the running time by using LSSVR and MKRVR to estimate the knee joint angle. Figure 3f compared the running time of different subjects generated by the MKRVR. It can be obtained from these figures that, whatever method is used, the running time increases as the whole of the sample size increases. The MKRVR has a significant advantage over the LSSVR in time, which can save five to ten cycles. The MKRVR generally takes less than 5 ms, while the LSSVR takes at least 4.5 ms. This further shows that MKRVR has a stronger generalization ability for small samples than LSSVR. MKRVR is more suitable for small sample regression training. These results may be due to the MKRVR based on the Bayesian framework, removing irrelevant points, according to automatic relevance determination, which greatly reduces the calculation of kernel function. To reduce the interference of abnormal parameters in the learning process, there needs to be a preprocessing method to generate a better input for MKRVR from the raw data.

Figure 4 and Figure 5 show the MAE and RMSE of different subjects when estimating the knee joint angle using surface EMG signals. As shown in Figure 4 and Figure 5, (a)–(e) compared MAE and RMSE of the estimation models of different sampling subjects under different sample sizes. The blue curve represents the MAE and RMSE obtained from the estimation model, established by LSSVR. The orange curve represents the MAE and RMSE obtained from the estimation model established by MKRVR. (f) indicates the MAE and RMSE changes in knee joint angle, which are estimated by two methods for different subjects when the sample size is 60 GCs (Where the A-value represents the original value, and the D-value represents the difference between the A-value of MAE and the RMSE obtained by two methods.). It can be seen, from the figure, that the changing trend of MAE and RMSE curves is the same. By contrast, RMSE is more obvious at the mutation position than MAE, which further illustrates the ability of RMSE to characterize abnormal characteristics. With the increase in sample size, the MAE and RMSE values gradually decrease.

According to the numerical description in Table 5, when the MKRVR was used, the best MAE, corresponding to different subjects, ranges from 1.95° to 4.64°. The best RMSE, corresponding to different subjects, ranges from 3.57° to 6.56°. When the LSSVR is used, the best MAE, corresponding to different subjects, is between 2.85° and 5.61°, and the best RMSE, corresponding to different subjects, ranges from 4.3° to 7.79°, which shows that the MKRVR method is better than the LSSVR method in estimating the knee joint angle. The MKRVR can continuously estimate the knee joint angle with a global MAE of 3.32° ± 1.37° and a RMSE of 4.86° ± 1.37°. These results seem to be large in comparison to the actual motion angle of the knee. It is acceptable for the application of controlling wearable robots because the actuators of robots have motion errors, and the wearer has both flexibility and tolerance to the small differences of several degrees. In addition, the estimation model, using MKRVR, has some hyperparameters on both the kernel functions and weight. Therefore, a better parameter set leads to a more accurate estimation model of MKRVR.

Figure 6 describes the comparison of model estimation accuracy when LSSVR and MKRVR are used. Figure 6a–e show the R^2^ of different subjects under the conditions of two methods. Figure 6f illustrates the R^2^ of different subjects with a marked stacked line chart when MKRVR is used. It illustrates that, when the two methods are used to estimate the knee joint angle, the estimation accuracy will increase with the increase in the sample size. From the analysis of the R^2^ change trend of the two methods, however, the sample size changes, and the results of the MKRVR method show obvious regularity. Since the LSSVR method is used, R^2^ will appear less than 0 when the sample size is less than 30 GCs. It means that there are errors in parameter or feature configuration during training. It indicates that, when the sample size is small, the feature learning ability of LSSVR is limited. The method cannot achieve ideal generalization ability. Therefore, the MKRVR method has more advantages than the LSSVR method in processing small sample estimation.

According to the information in Table 6, when the LSSVR is used, the best R^2^ values, corresponding to different objects, are (S1, 0.743), (S2, 0.864), (S3, 0.913), and (S4, 0.794), and (S5, 0.841). When MKRVR is used, the best R^2^ values, corresponding to different subjects, are (S1, 0.821), (S2, 0.921), (S3, 0.951), (S4, 0.871), and (S5, 0.911). The results showed that LSSVR can continuously estimate the knee joint angle with a global R^2^ of 0.8308 ± 0.09, and the MKRVR can continuously estimate the knee joint angle with a global R^2^ of 0.8946 ± 0.07. The accuracy of model estimation established by the latter is better than the former by comparison. Once the sample size is more than 20 GCs, the subsequent changes in the estimation accuracy would be stabilized. It means that, when the sample size increases to a certain value, the larger sample size would further enhance the estimation accuracy.

According to the above estimation results, the comparison curve of the estimated knee joint angle and the true knee joint angle of subject 3 is selected to illustrate the estimation accuracy of the two methods with different sample sizes. This can be seen in Figure 7. From the figure, the coincidence degree of the estimated knee joint angle curve and the true value curve when using different methods can be seen. Figure 7 selects the estimation results when the sample sizes are 1 GC, 5 GCs, 10 GCs, 20 GCs, 30 GCs, 40 GCs, 50 GCs, and 60 GCs to illustrate the corresponding relationship between the estimated knee joint angle and the increased of the sample size. It can be seen that the changing trend of the estimated knee joint angle corresponds to the changing trend of other evaluation criteria. The estimation accuracy will be improved with the increase in the sample size as a whole.

## 5. Discussion

In this study, a novel estimation approach based on MKRVR was proposed to construct the mapping relationship between the knee joint angles and sEMG signals. According to the results, the proposed approach improves the angle estimation in comparison with the LSSVR and standard RVR with the Gaussian RBF kernel. The sampling size is positively correlated with the estimation accuracy, and the data partitioning method based on the gait cycle avoids the impact of individual differences, such as gait speed, stride, etc. The AF is used to reconnect each GC as a complete dataset. The estimation accuracy of MKRVR is slightly better than the standard RVR with Gaussian RBF kernel. The reason may be related to the dataset quality, hyperparameters, muscle synergies, etc, but the estimation accuracy has been further improved than LSSVR. This result showed that MKRVR can construct a better estimation model, depending on its relevance.

According to the above results, since the sample size increases to a certain value, the running time shows a sudden change, and the uncertainty of the running time increases with the sample size increase. On the premise of ensuring estimation accuracy, selecting a reasonable sample size is more meaningful for decreasing running time and improving the practicability of sEMG signals on the online control of wearable robots.

In comparison, the estimation process using MKRVR is stable, and, even if 1 GC is used as the training sample, good results can still be achieved. When the sample size is greater than 10 GCs, the MAE and RMSE values are significantly reduced. While the estimation process using LSSVR is relatively variable, only when the sample size is greater than 30 GCs will the MAE and RMSE values show a regular decrease. The above results can explain that the MKRVR can quickly realize the generalization of the regression process under a limited sample size.

This study aimed to lay the foundation for future development in the application of human–robot collaboration control and construct the HRI system based on sEMG to predict human motion intention accurately. However, sEMG is unstable, and only the investigated cases of unimpaired persons were considered for normal walking. Hence, the exceptions from the input data sets must be removed before estimation. Otherwise, the estimation accuracy shows a larger uncertainty variation. Thus, more subjects will be invited to participate in the research to validate the composited approaches further.

From the coincidence degree between the estimated knee angle curve and the true value curve in the figure, it can be seen intuitively that MAE and RMSE cannot be indicated. The knee angle curve estimated by LSSVR fluctuates more, especially in the gait-switching stage when the number of sampling points is 60. However, MKRVR reduces the abnormal fluctuation at this stage and improves the coincidence degree with the true value. The results of the LSSVR are more unstable, relatively, and the results of the MKRVR have estimations similar to the raw value. That is, MKRVR has a better error tolerance.

Of course, different machine learning methods have different advantages, which depend on the settings of hyperparameters, the size of datasets, the use of dimension reduction methods, and the requirements of application scenarios. In this study, the MKRVR method demonstrated excellent regression estimation performance in a small sample size. Although the MKRVR performs brightly better than LSSVR, compared to standard RVR, it performs slightly better, and this may be related to the kernel weight of multiple kernels, as the kernel weight is set based on prior knowledge and manual verification. According to the results, improving the estimation accuracy should be performed by collecting more sEMG signals from relevant muscles and adding the preprocessing of sEMG signals based on muscle synergy.

## 6. Conclusions and Future Work

In this study, a novel estimation model of MKRVR was proposed to estimate the knee joint angle based on sEMG and study their relationship with the HRI of wearable robots. In the preprocessing of the raw sEMG signals, AF is used to extract the sEMG features of sample points in different gait cycles to improve the consistency of input data. The comparison between MKRVR and LSSVR is proposed to verify the usefulness of the proposed method.

According to the results, MKRVR performs better than RFPCA\RVR\RF\LSSVR in the estimation of small samples, and MKRVR and RVR have more advantages in execution time. The MKRVR performs better than the LSSVR in the estimation of the knee joint angle, mainly in the following aspects: less running time, higher estimation accuracy, and better stability in small sample estimation under the same sample size. The results showed that the MKRVR can continuously estimate the knee joint angle, with a global MAE of 3.27° ± 1.2°, a RMSE of 4.81° ± 1.37°, and a R^2^ of 0.8946 ± 0.07. This concludes that MKRVR is suitable for knee joint angle estimation from the sEMG signals with high accuracy and requiring minimal time. The MKRVR will be used to promote the safety and easiness of sEMG in the application of recognizing the wearer’s motion intentions while controlling wearable robots. These results also demonstrate the potential for this method to achieve rapid online intention recognition regarding human–robot collaboration control.

Future work on improving the estimation accuracy will be performed by using an intelligent optimization algorithm to optimize the hyperparameters of the kernel function in the model. Future research will extract sEMG signals from more than five muscles to analyze the muscle synergies and the relationship between the sEMG and joint movement. The composited groups of different muscles and different features will be selected as the inputs to train the regression model. Furthermore, the proposed method in this work will be verified on the public dataset, such as the CSL-SHARE dataset [38]. Finally, this method will be applied in the control of wearable extra robotic limbs.

## Figures and Tables

**Table 1:** The information of participants.

Participants	Age	Height/cm	Mass/kg	Thigh Diameter/mm	Shank Diameter/mm
S1	24	175	61	45	34
S2	27	179	74	54	40
S3	24	183	75	50	39
S4	24	180	68	43	35
S5	22	186	80	51	40

**Table 2:** The sample size of different subjects in a gait cycle.

S	1#	2#	3#	4#	5#
n	129±9	127±6	126±7	131±6	122±7

**Table 3:** The key parameters of the algorithms.

Parameters	*d*	*γ*	*c*	*K_gaussian_*	*K_polynomial_*	*K_sigmoid_*
Value	0.02	[0.01, 0.005]	0.01	0.8	0.17	0.03

**Table 4:** The average accuracy of different methods.

	R2_Score	RMSE	MAE	Time
RVR	0.8881 ± 0.05	5.17° ± 1.34°	3.58° ± 1.3°	1.2 ± 0.9 ms
LSSVR	0.8308 ± 0.09	6.19° ± 1.58°	4.25° ± 1.2°	13.31 ± 0.5 ms
RF	0.8698 ± 0.05	5.36° ± 1.18°	3.41° ± 1.1°	2.97 ± 0.05 ms
RFPCA	0.8924 ± 0.04	4.94° ± 1.26°	3.24° ± 0.6°	2.99 ± 0.1 ms
MKRVR	0.8946 ± 0.07	4.81° ± 1.37°	3.27° ± 1.2°	1.9 ± 0.2 ms

**Table 5:** The results of LSSVR and MKRVR.

	LSSVR						MKRVR					
	Time Range	Δ*t*	MAE Range	Δ*d*	RMSE Range	Δ*d*	Time Range	Δ*t*	MAE Range	Δ*d*	RMSE Range	Δ*d*
S1	[13.69, 26.27]	12.58	[5.61, 17.03]	11.42	[7.79, 24.29]	16.5	[2.13, 4.86]	2.73	[4.64, 9.13]	4.49	[6.56, 11.66]	5.1
S2	[5.43, 20.77]	15.34	[4, 13.07]	9.07	[6.21, 16.89]	10.68	[0.58, 1.07]	0.49	[3.01, 6.6]	3.59	[4.46, 9.56]	5.1
S3	[5.11, 19.93]	14.82	[2.85, 14.17]	11.32	[4.3, 31.16]	26.86	[0.93, 2.69]	1.76	[1.95, 6.09]	4.14	[3.45, 7.48]	4.03
S4	[4.91, 18.11]	13.21	[4.69, 27.65]	22.96	[6.71, 39.6]	32.89	[0.53, 2.35]	1.82	[3.63, 8.05]	4.42	[5.02, 10.57]	5.55
S5	[4.53, 15.83]	11.3	[4.09, 29.53]	25.44	[5.79, 39.32]	33.53	[0.8, 2.81]	2.01	[3.37, 8.53]	5.16	[4.54, 10.48]	5.94

**Table 6:** The R2_scores of different subjects with LSSVR and MKRVR.

	Method	LSSVR	MKRVR
Subject			
S1		[0.555, 0.743]	[0.588, 0.816]
S2		[0.413, 0.862]	[0.668, 0.921]
S3		[0.458, 0.913]	[0.689, 0.951]
S4		[0.439, 0.794]	[0.467, 0.871]
S5		[0.311, 0.841]	[0.217, 0.911]

## Data Availability

The data presented in this study are available on request from the corresponding author. The data are not publicly available due to the data also forms part of an ongoing study.
